# Preharvest Treatment with Oxalic Acid Improves Postharvest Storage of Lemon Fruit by Stimulation of the Antioxidant System and Phenolic Content

**DOI:** 10.3390/antiox10060963

**Published:** 2021-06-15

**Authors:** Vicente Serna-Escolano, María José Giménez, Salvador Castillo, Juan Miguel Valverde, Domingo Martínez-Romero, Fabián Guillén, María Serrano, Daniel Valero, Pedro Javier Zapata

**Affiliations:** 1 Department of Food Technology, EPSO, University Miguel Hernández, 03312 Alicante, Spain; vicente.serna02@goumh.umh.es (V.S.-E.); maria.gimenezt@umh.es (M.J.G.); scastillo@umh.es (S.C.); jmvalverde@umh.es (J.M.V.); dmromero@umh.es (D.M.-R.); fabian.guillen@umh.es (F.G.); daniel.valero@umh.es (D.V.); 2 Department of Applied Biology, EPSO, University Miguel Hernández, 03312 Alicante, Spain; m.serrano@umh.es

**Keywords:** lemon tree, oxalic acid, antioxidant enzymes, total phenolic content, preharvest, postharvest

## Abstract

Lemon trees (*Citrus limon* (L.) Burm. F) were treated monthly with oxalic acid (OA) at 0.1, 0.5, and 1 mM from initial fruit growth on the tree until harvest in2019. The experiment was repeated in 2020, with the application of OA 1 mM (according to the best results of 2019). In both years, fruit from OA-treated trees and the controls were stored for 35 days at 10 °C. Results showed that all treatments reduced weight loss (WL) and maintained higher firmness, total soluble solids (TSS), and total acidity (TA) than in the controls. Meanwhile, colour (hue angle) did not show significant differences. The activity of antioxidant enzymes, catalase (CAT), ascorbate peroxidase (APX), and peroxidase (POD) in the flavedo of the fruit from the OA-treated trees was higher than in the controls at harvest and after 35 days of storage. Similarly, the total phenolic content (TPC) in the flavedo and juice of the fruit from the OA-treated trees were higher than in the controls. The increase in the activity of the antioxidant enzymes and TPC started with the first preharvest OA treatment and were maintained during fruit development on the tree until harvest. Preharvest OA treatments enhanced the antioxidant system of the lemon fruits, reducing the postharvest incidence of decay. Thus, OA could be a useful tool to increase the quality and functional properties of lemon fruits.

## 1. Introduction

The lemon tree (*Citrus limon* (L.) Burm. F) is an evergreen plant belonging to the *Rutaceae* family, mainly cultivated in countries of the tropical and subtropical regions. Spain is positioned as one of the main producer countries of lemon fruit worldwide, together with Argentina, Mexico, China, and India [1]. Lemons are highly appreciated by consumers around the world because of their flavour and appearance [2], and are widely used for culinary purposes, such as juice, slices accompanying beverages, or as flavouring (pulp and peel). In addition, lemon fruit has a high content of bioactive compounds, the most important ones being carotenoids, ascorbic acid (AsA), and flavonoids, mainly eriocitrin and hesperidin, which determine its biological activity [3,4]. These attributes are dependent on different factors such as management of the crop, the growth and fruit ripening stages, and rootstock, among others [5,6]

Lemons have a limited storage life, mainly due to softening, colour changes, acidity losses, and decay [2] as well as the oxidation of bioactive compounds. The content of AsA and antioxidant activity in lemon juice are affected by the ripening stage at harvest, as the higher initial concentration of antioxidant compounds could delay quality loss during storage since low antioxidant activity can produce an imbalance in reactive oxygen species (ROS), which would damage cell membranes and accelerate fruit senescence [7]. In this sense, the combination of cold storage and some postharvest treatments have been shown to be useful in delaying the postharvest ripening process [8]. Citrus fruit decay is mainly due to *Penicillium digitatum*, *Penicillium italicum*, and *Geotrichum citri-aurantii,* although the last one is less common. Additionally, the decay is related to mechanical damage on the fruit surface and high humidity during the harvest, storage, and fruit maturity stages [2]. The main fungicides to control lemon fruit decay are or-tho-phenylphenol (OPP), imazalil (IMZ), and thiabendazole (TBZ); however, many of them are being limited by legislation due to risks to human health. 

One of the emerging strategies to delay the postharvest losses during storage is the use of preharvest treatments with natural elicitor compounds, which stimulate a tree response, leading to the maintenance of fruit quality through delaying the maturation process during cold storage. Among these elicitors, oxalic acid (OA) is a naturally organic acid present in plants, fungi, and mammals, playing different roles for each organism [9]. Specifically in fruits, it has been reported that preharvest treatments with OA have positive effects in delaying senescence and preserving the quality in sweet cherries [10], peaches [11], and pomegranates [12]. Although OA’s mechanism of action has not been elucidated, postharvest treatments with OA in mangoes have been related to the scavenging of the accumulation of reactive oxygen species (ROS) [13], and the maintenance of high contents of AsA and high levels of ATP in postharvest OA-treated kiwifruit [14]. Increases in the ratio of unsaturated/saturated fatty acids and the content of glucose and fructose have been reported in peaches and apricots, respectively, improving chilling injury tolerance in postharvest OA-treated samples [15,16]. Moreover, postharvest treatments with OA in melons and jujube were related to a reduction of decay due to an increase in the activity of antioxidant enzymes related to the resistance to pathogens, including peroxidase (POD), ascorbate peroxidase (APX), superoxide dismutase (SOD), and catalase (CAT) [17,18]. However, to the best of our knowledge, there is no information available in the literature regarding the effect of OA treatments on lemon fruit quality at harvest or during storage. OA is used as a pesticide to control the Varroa mite in honeybee combs. Recently, the FDA finalized a regulation establishing an exemption from the requirement of a tolerance for of OA residues in honey and honeycomb, concluding that OA is safe for the general U.S. population, or any population subgroup, including infants and children.

The aim of this study was to evaluate the effect of preharvest treatments with OA at 0.1, 0.5, and 1 mM on the antioxidant enzymes CAT, APX, and POD in the flavedo and the total phenolic content in juice and flavedo of lemon fruit during on-tree development. Furthermore, the effect of OA treatments on lemon quality traits over 35 days of postharvest storage was measured. Finally, the role of the increased antioxidant system by OA treatments and the maintenance of postharvest quality of lemon fruits is discussed.

## 2. Material and Methods

### 2.1. Field Conditions and Experimental Design

Commercial fields located in Cartagena (Murcia, Spain) were used to perform the experiments in 2019 and 2020 using lemon (*Citrus limon* (L.) Burm.f) ‘Fino’ grafted on *Citrus macrophylla* rootstock, under standard agricultural practices that include foliar sprays of sulphur or copper for fungal control during periods with high field humidity. The trees were planted at 4 × 5 m, with the ‘Fino’ trees being 8 and 9 years old in the 2019 and 2020 assays, respectively. Field experiments were performed at random using four blocks of five trees for each treatment. In 2019, assay ‘Fino’ trees were treated five times with 0.1, 0.5, and 1.0 mM of OA at monthly intervals, starting after physiological fruit drop (abscission of green, young fruits due to physiological reasons) in July and finishing three days before harvest (13th December). In the 2020 experiment, according to the best results obtained in 2019, the concentration of 1.0 mM OA was chosen and applied as it was in the 2019 experiment, and the fruit was harvested on 11st December. For all cases, 5 L of OA (Sigma-Aldrich, Madrid, Spain) solutions containing 0.5% Tween 20 as a surfactant were sprayed on each tree. Control trees were sprayed with aqueous solutions containing 0.5% Tween-20. The fruit was harvested at the commercial ripening stage based on size (55 mm of diameter) and peel colour (light green). For the postharvest experiments in 2019 and 2020, 60 fruits (homogeneous in size and colour) from each replicate and treatment field were taken, divided into six lots of ten fruits, and stored at 10 °C at a relative humidity of 85%. One lot of ten fruits for each replicate and treatment was sampled at random after 0, 7, 14, 28, and 35 days of cold storage for analytical determinations. In addition, in the 2020 experiment, two lemon fruits were picked from each tree after 3 days of treatment 1 (T1) and treatment 5 (T5); after 3, 4, 5, and 6 days of treatment 2 (T2) and treatment 3 (T3); and after 3, 4, 5, 8, 11, 15, and 22 days of treatment 4 (T4). The fruits were peeled and squeezed, and the peels and juice of the 10 fruits of each replicate were mixed and stored at −80 °C until the total phenolics and the activity of the antioxidant enzymes were measured.

### 2.2. Physiological and Quality Parameters

Each individual fruit was weighed at day 0 and after each sampling date during storage, and weight loss (WL) was expressed as a percentage (%). Firmness was measured individually in each fruit by using a TX-XT2i texture analyser (Stable Microsystems, Godalming, UK) coupled to a steel plate that caused a deformation of 5% of the fruit diameter and was expressed as N mm^−1^. On the other hand, to measure the respiration rate, each fruit was placed in a 0.5 L glass jar for60 min at room temperature. One mL of the holder atmosphere was withdrawn, and CO_2_ was quantified in a gas chromatograph (Shimadzu 14B-GC) coupled to a thermal conductivity detector [19]. The respiration rate was expressed as mg of CO_2_ kg^−1^ h^−1^. The colour was measured at three points of the equatorial perimeter using the Minolta colorimeter (CRC200; Minolta, Osaka, Japan) and was expressed as hue angle. All of these parameters were measured in the ten fruits of each replicate, and results were expressed as mean ± SE. The juice of the 10 fruits from each replicate was then mixed and used to measure total soluble solids (TSS) and titratable acidity (TA) in duplicate in each sample, and the peels were mixed and frozen in liquid nitrogen. The TSS in the lemon juice was determined with a digital refractometer (Hanna Instruments, Woonsocket, RI, USA), and the TA was determined by the titration of 0.5 mL of juice diluted in 25 mL of distilled water with NaOH 0.1 mM until pH 8.1 using an automatic titrator (785 DMP Titrino; Metrohm, Herisau, Switzerland). The results (mean ± SE) were expressed as g 100 mL^−1^. The incidence of decay was determined for each sampling day by identifying the lemons with disease symptoms [20] using the following formula:

Decay (%) = (decayed fruits/total evaluated fruits) × 100(1)

### 2.3. Activity of the Antioxidant Enzymes

Extracts to measure ascorbate peroxidase (APX), catalase (CAT), and peroxidase (POD) were obtained by homogenizing 2 g of frozen flavedo tissue with 10 mL of potassium phosphate buffer 50 mM, pH 7, containing 1% (*w*/*v*) polyvinylpyrrolidone and 1.0 mM of ethylenediamine tetraacetic acid. The extracts were centrifuged at 10,000× *g* for 20 min at 4 °C, and the APX, CAT, and POD activities were quantified in duplicate in each extract as previously reported [21]. Enzymatic activities were calculated as units of enzyme activity (U min^−1^ g^−1^). One enzyme unit (U) was defined as a 0.01 decrease in absorbance at 290 nm min^−1^ for APX, a 0.01 increase of absorbance at 470 nm min^−1^ for POD and, a 0.01 decrease of absorbance at 240 nm min^−1^ for CAT.

### 2.4. Total Phenolic Quantification

The total phenolics were measured by homogenizing 2 g of frozen flavedo with 15 mL of water: methanol (2:8, *v*/*v*) containing 2.0 mM NaF. After centrifugation of the extracts at 10,000× *g* and 4 °C for 20 min, the total phenolic content (TPC) was measured in duplicate in each flavedo sample by using the Folin–Ciocalteau reagent, as previously reported [21]. The total phenolics in the juice were measured directly using the centrifugated juice in duplicate for each replicate and treatment. Results (mean ± SE) were expressed as mg of gallic acid equivalent to 100 g^−1^ fresh weight.

### 2.5. Statistical Analysis

The results obtained were expressed as the mean ± SE of four randomized replicates. Data were subjected to analysis of variance (ANOVA) and a multiple comparison with the Fisher LSD method was carried out to find significant differences (*p* < 0.05) among the treatments, using SPSS, version 22 (IBM Corp., Armonk, NY, USA).

## 3. Results

### 3.1. Physiological and Quality Parameters (2019)

Weight loss increased during cold storage (Table 1), although the OA treatment induced significantly lower weight loss (*p* < 0.05), with compared to the lemons from the control trees for all assayed doses (0.1, 0.5 and 1 mM). After 35 days of cold storage, lemons from the 1 mM treated trees had 40% lower WL than the controls, OA 1 mM being considered the best treatment in terms of reduced physiological WL. Conversely, fruit firmness decreased during cold storage, although this decrease was delayed by both the 0.5 and 1 mM OA treatments, with the most significant effect (*p* < 0.05) being found at day 35 of storage for the fruit from the 0.5 mM OA treatment (a 8% higher firmness retention), while no significant effect was observed for 0.1 mM treatment (Table 1).

At harvest, the respiration rate was lower in lemons from the OA-treated trees at 0.1, 0.5, and mM (5%, 14%, and 30% lower, respectively), than in fruits from the control trees, with these differences being maintained after 35 days of cold storage. At harvest, the OA 1 mM treatment increased TSS (a 7%) while the OA 0.1 and 0.5 mM treatments did not show significant differences (*p* < 0.05) with respect to lemons from the control trees. The TSS decreased during cold storage, although these differences in the TSS at harvest were maintained during storage. A similar effect was observed for TA, which decreased during cold storage, but lemons from the 1 mM OA-treated trees had significantly a higher TA than the other treated and untreated fruits (Table 1). On the other hand, the hue angle at harvest of fruit from the 0.1 mM treated trees showed no significant values according to statistical analysis (more yellow) than fruit from the trees treated with OA 1 mM and the controls. The differences found at harvest were maintained during cold storage. There were no significant differences (*p* < 0.05) between fruits from trees treated with OA 1 mM and the controls during the assay. However, the hue angle decreased during storage in all fruits, independent of the treatments (Table 1).

### 3.2. Antioxidant Systems during Cold Storage (2019)

At harvest, APX, CAT, and POD activity in the flavedo of the lemons from the OA-treated trees were higher than in the controls, OA at 1 mM being the most effective treatment in enhancing these antioxidant activities. After 35 days of storage, the differences among treatments were reduced, although the activity of APX, CAT, and POD was 1.9, 2.6, and 1.7-fold higher in the flavedo of the fruit from the 1 mM OA-treated trees, respectively, than in the controls (Table 2). TPC at harvest in the flavedo of the fruit from the trees treated with OA at 0.1, 0.5, and 1 mM were 30%, 52%, and 71% higher than in the controls, respectively. The TPC in the juice were significantly (*p* < 0.05) increased by the 0.5 and 1 mM treatments. The TPC increased in fruits from the treated and untreated trees during cold storage in the flavedo and juice, TPC still being higher for OA treatments at 0.5 and 1 mM than in controls.

Taking into account that quality parameters, such as WL, firmness, TA, and TSS as well as the TPC and the activity of the antioxidant enzymes, were found in higher levels at harvest and maintained at higher levels during storage in the fruit from the 1 mM OA treated trees as compared with those from the 0.1 and 0.5 mM doses, this concentration was selected to perform the 2020 experiment.

### 3.3. Antioxidant Systems during Fruit Development and Cold Storage (2020)

**Figure 1:**
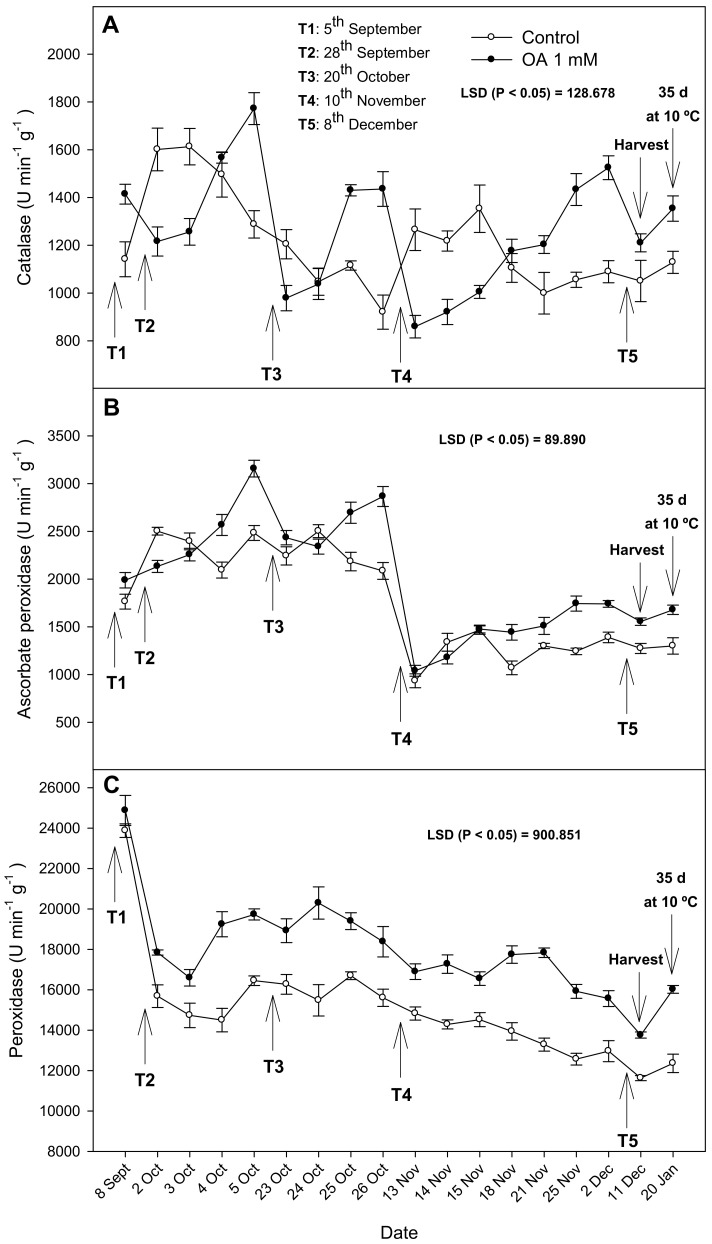
The effect of oxalic acid (OA) treatments on catalase (CAT), ascorbate peroxidase (APX), and peroxidase (POD) activity on the flavedo of lemon fruit during growth and ripening on the tree (**A**–**C**, respectively), at harvest, and after 35 days of storage at 10 °C in the 2020 experiment. Data are the the mean ± SE of the four replicates. LSD show significant differences at *p* < 0.05. T1, T2, T3, T4, and T5 show the dates of the treatments.

**Figure 2:**
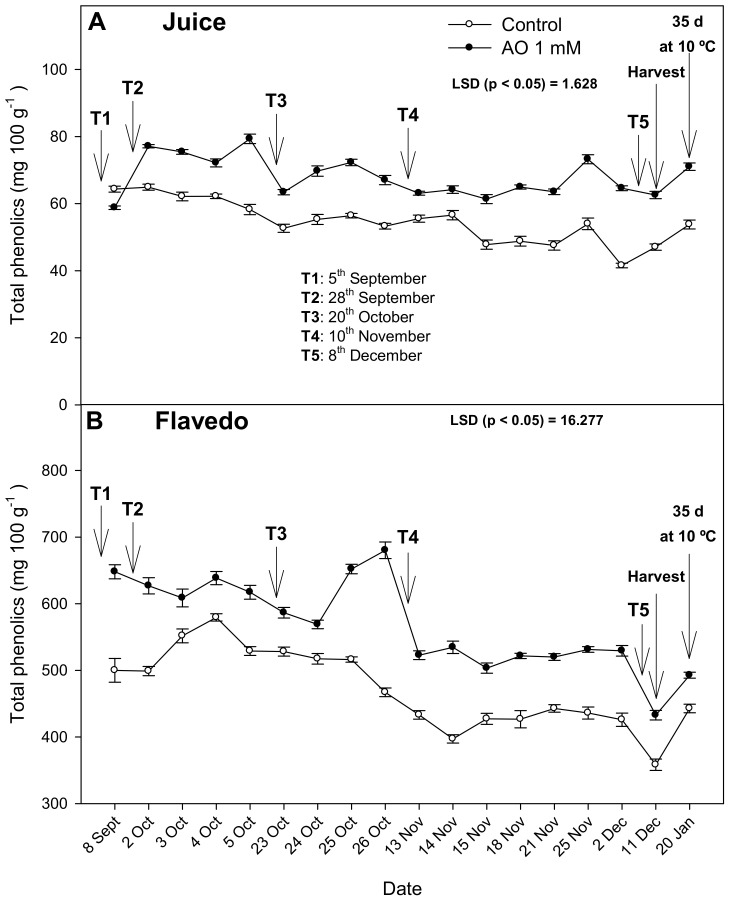
Total phenolic content in juice (**A**) and flavedo (**B**) during fruit growth and ripening on the tree, at harvest, and after 35 days of storage at 10 °C of lemons from the control and oxalic acid (OA) treated trees in the 2020 experiment. Data are the mean ± SE of four replicates. LSD show significant differences at *p* < 0.05. T1, T2, T3, T4, and T5 show the dates of the treatments.

In 2020, a more detailed study was developed, measuring the activity of the antioxidant enzymes CAT, APX, and POD in the flavedo of the lemon fruits treated with OA 1 mM during their development on the tree and after 35 days of cold storage (Figure 1). The TPC in the flavedo and juice were also determined during fruit development (Figure 2). The activity of CAT enzymes changed during fruit development on the tree. Since the first sampling on 8th September (three days after the first treatment), significant differences (*p* < 0.05) were found between the fruit treated with OA and the control fruit. However, in the second treatment, the maximum enzymatic activity was measured from the fourth day and was 40% higher in the fruit treated with OA than the control fruit (Figure 1). Similar to the previous treatment, the third application showed that the OA treatment had increased CAT activity by 45% compared to the control fruit. However, after four days of T1, T2, and T3, CAT activity decreased, reaching lower levels than in the controls. From the fourth treatment, CAT activity increased, and the major activity peak was achieved after 21st days, being 50% higher in the OA treated fruit than the control fruit. The APX enzyme had similar trend as CAT, and it reached the maximum activity four days after T1 and T3 and after 14 days of T4, with an increase of 45% compared to the untreated fruit (1243 ± 34 U min^−1^ g^−1^). On the other hand, the POD enzyme had different behavior, as it had a significant increase (*p* < 0.05) after the second treatment and was maintained at higher levels than in the control fruits during the development of the fruit on the tree (Figure 1).

The lemon fruits were harvested on 11th December, and the flavedo of the lemons from the OA-treated trees showed significantly higher (*p* < 0.05) activities of CAT, POD, and APX (ca. 20%) than the controls. After 35 days of storage, fruits from the control trees showed CAT activity of 1128 ± 46 U min^−1^ g^−1^, APX of 1300 ± 85 U min^−1^ g^−1^, and POD of 12,360 ± 456 U min^−1^ g^−1^, and they were significantly higher (*p* < 0.05) in the fruit from the trees treated with OA 1 mM, by 20%, 30%, and 29%, respectively than in the controls (Figure 1).

The total phenolics content in juice increased 30% from T2 in lemons from the OA-treated trees compared to the controls, differences that remained at similar levels during the whole fruit development until harvest. However, the TPC in the flavedo showed different behavior because a decreasing trend was observed in the lemons from the control and treated trees during fruit development. However, it is worth noting that TPC in the flavedo was significantly (*p* < 0.05) increased by OA treatment from the first treatment until harvest, the highest differences being found after T3 treatment (45% increase). During storage, TPC increased in all fruits, but after 35 days of storage, values were 33% and 11% higher in flavedo and juice, respectively, in fruits from the OA-treated trees than in the controls (Figure 2).

### 3.4. Physiological and Quality Parameters (2020)

**Figure 3:**
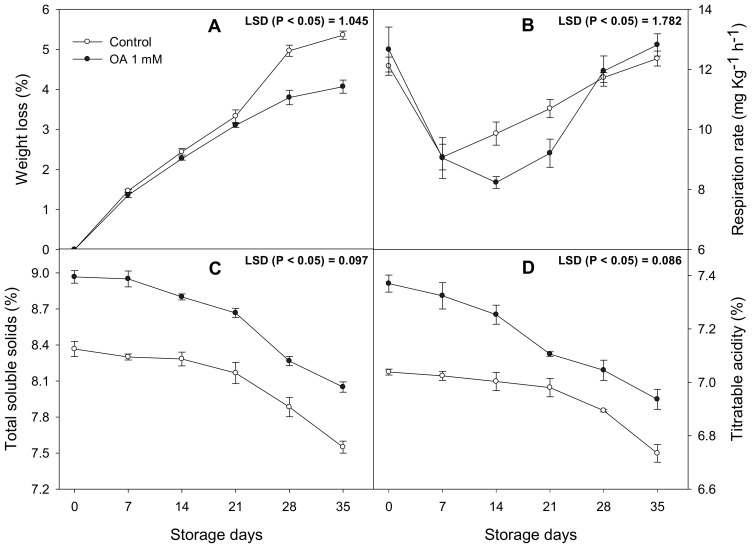
The effect of oxalic acid (OA) treatments in (**A**) weight loss (%), (**B**) respiration rate (mg kg^−1^ h^−1^), (**C**) total soluble solids (mg 100 g^−1^), and (**D**) titrable acidity (mg 100 g^−1^) during 35 days of storage at 10 °C in the 2020 experiment. Data are the mean ± SE. LSD show significant differences at *p* < 0.05.

The fruit quality parameters were affected by OA treatment, as they were in the 2019 assays. WL was significantly lower (*p* < 0.05) in the fruit from the 1 mM OA treated trees than in the controls from 21 to 35 days of cold storage (Figure 3A). The respiration rate was significantly reduced in the treated fruit at 14 and 21 days of storage. The greatest respiration rate difference was on day 14, with a decrease of 30% in the OA treated fruit compared to the controls (Figure 3B).

**Figure 4:**
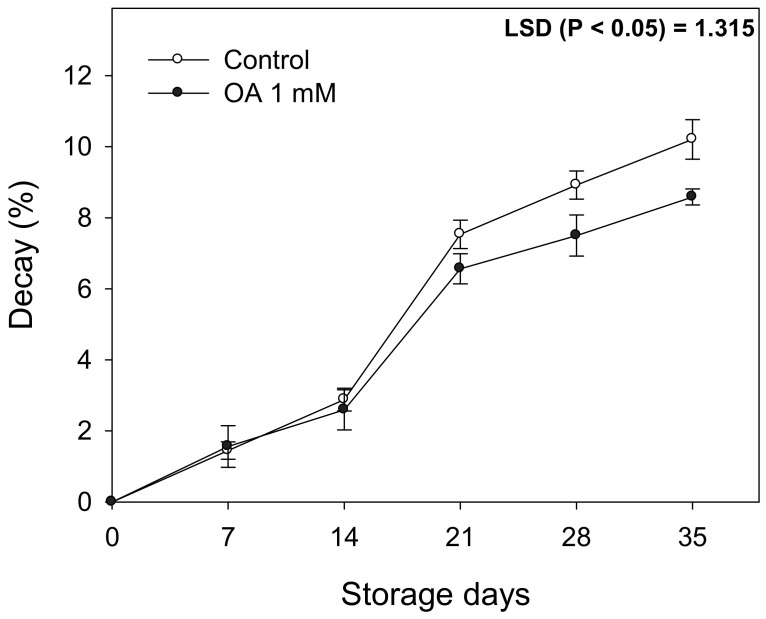
The incidence (%) of decayed lemons from the trees treated with oxalic acid (OA) 1 mM and in the controls. Data are the mean ± SE. LSD show significant differences at *p* < 0.05.

The total soluble solids and TA in the fruit from OA-treated trees increased 10% and 3%, respectively, compared to the controls, and these differences were maintained during storage (Figure 3C,D). Finally, the decay incidence was evaluated as the percentage of fruit showing symptoms of fungal decay since previous results (2019) confirmed the effect of OA-treatments on shelf life. Results showed that from 21 days until to the end of storage, fruit from the 1 mM OA treated trees had lower decay incidence than the controls, having the highest differences after 35 days of storage with a 20% decrease (Figure 4).

## 4. Discussion

Commercial procedures regulate the marketing of lemons by increasing requirements regarding quality parameters. In addition, there is currently a growing interest in replacing traditional treatments with others with a lower environmental impact. Previous results in fruits such as cherries [10] or plums [22] established that preharvest treatments with OA could delay fruit quality losses during cold storage. In lemon fruits, OA treatments considerably reduced the respiration rate, which could be responsible for the lower weight loss and higher firmness observed in the fruits from the treated trees than in the controls in the 2019 (Table 1) and 2020 (Figure 3) experiments. These results were consistent with previous results in pomegranates [12], mangoes [23], and artichokes [24]. Differences between the treated and untreated fruits with respect to weight loss and firmness could be attributed to the effect of OA on the stabilization and maintenance of cell integrity and on the reduction of the hydrolysis of cell wall components in the mesocarp [8]. Recent assays reported that postharvest treatments with OA in asparagus preserved quality by reducing the respiration rate during cold storage [25], which would indicate a suppressive effect on metabolic activity, leading to a lower consumption of TSS and organic acids [26]. At harvest, OA treated fruit had higher TSS and TA than untreated fruit in 2019 (Table 1) and 2020 (Figure 3C), which could be due to an increase in the photosynthetic rate that promotes sugar and organic acid accumulation during fruit development on the tree. However, these differences were maintained during cold storage due to the reduced hydrolysis of sugars and organic acids, maybe related to a lower respiration rate.

In the 2019 experiment, antioxidant activity at harvest of the APX, CAT, and POD enzymes was higher in fruit treated with OA than in untreated fruit, and these effects were maintained over 35 days of storage (Table 2). In 2020, it was decided to carry out a deeper assay comparing the fruit treated with the best concentration of OA (1 mM), according to the 2019 results. APX and POD activity decreased during fruit development on the tree in both treated and untreated lemons (Figure 1B,C). However, CAT activity was more stable from the beginning to the end of the assay (Figure 1A). These differences may be related to the balance between the production and elimination of H_2_O_2_, which could be due to the important role that OA plays in suppressing oxidative damage [13]. However, it is important to note that all of those antioxidant enzymes were enhanced by OA from the first application, although an accumulative effect due to repeated applications could not be observed.

Antioxidant activity of the APX, CAT, and POD enzymes increased during cold storage in all samples, however, the increase in the fruit treated with OA was significantly higher than in the untreated fruit. CAT, POD, and APX are enzymes that have a wide distribution in taller plants and have a very specific function in detoxifying ROS [27]. Preharvest OA treatments would be able to maintain higher activity of CAT and POD for a longer time, ensuring better control of ROS balance in cells.

On the other hand, TPC were also increased by the OA treatments in the juice and flavedo in both years. In lemon fruit, the main phenolic compounds are the flavonones hesperidin and eriocitrin, which, together with vitamin C, determine most of non-enzymatic antioxidant activity [26]. The TPC in the flavedo decreased during fruit development on tree, which could be attributed to the oxidation of polyphenols by the polyphenoloxidase enzyme during the fruit ripening process [28]. TPC in the flavedo and juice increased significantly in fruit from the OA-treated trees and in the controls during storage, although the higher levels observed at harvest as a consequence of the OA treatments were maintained until the end of storage (Figure 2). These results were consistent with previous reports in mangoes [29], pomegranates [30], and plums [22], where OA treatments promoted the accumulation of TPC during cold storage due to the activation of phenylalanine ammonium lyase (PAL), a key enzyme of the phenylpropanoid pathway.

Preharvest OA treatment decreased the accumulated decay incidence in lemon fruit during cold storage (Figure 3E). This effect could be explained by the enhanced capacity of antioxidant enzymes found in the fruits treated with OA as well as to the OA effect of strengthening the cell wall and promoting the expression of genes in plant defense responses [14]. Lemon fruit phytopathogenic fungi such as *Penicillium digitatum* and *Penicillium italicum* are capable of altering oxidative balance, destabilizing the cell wall [31]. In this sense, it has been reported that *P. digitatum* induces the synthesis of secondary metabolites, such as flavonones, in citrus fruit through the stimulation of the phenylpropanoid pathway [32]. Moreover, there is evidence that indicates the importance of primary metabolism and the ability to generate ATP in the resistance of fruits to the pathogen [33]. Carbon availability is essential in many defense responses since in many cases, it requires the synthesis of biomolecules, such as the flavonones mentioned above. These results could establish that fruits treated with OA would have a greater availability of sugars and organic acids as a carbon source to obtain energy to induce defense mechanisms [34].

## 5. Conclusions

All preharvest treatments with OA showed a good effect on the quality parameters measured at harvest and during cold storage in 2019. However, treatment with 1mM OA demonstrated higher effectiveness compared to 0.1 and 0.5 mM, leading to lower weight loss, respiratory rate, higher firmness, TSS, and TA. Regarding bioactive compounds, the treated fruit showed the highest phenolic content and maximum activity of antioxidant enzymes (APX, CAT and POD), confirming 1 mM OA treatment as the best treatment. Therefore, the following year, the effects of the treatment with 1 mM OA were confirmed, showing lower quality losses and improvement in the shelf life of lemons during marketing and significantly reduced the appearance of decay, which could be attributed to enhanced antioxidant systems.

## Figures and Tables

**Table 1:** The effects of oxalic acid (OA) at 0.1, 0.5, and 1 mM preharvest treatments on weight loss (%), firmness (N mm^−1^), respiration rate (mg kg^−1^ h^−1^), total soluble solids (° Brix), total acidity (g 100 g^−1^), and colour (hue angle) over35 days of storage at 10 °C in the 2019 experiment. Data are the mean ± SE of four replicates. Different lowercase letters in the same column indicate significant differences (*p* < 0.05) among the treatments for each sampling date.

Storage	Treatment	Weight	Firmness	Respiration	Total Soluble	Titratable	Colour
Days		Loss		Rate	Solids	Acidity	
	**Control**		9.51 ± 0.30 a	15.72 ± 0.38 a	7.53 ± 0.09 b	6.67 ± 0.08 b	91.23 ± 0.50 a
0	**OA 0.1**		9.21 ± 0.18 a	14.85 ± 0.21 a	7.37 ± 0.07 c	6.71 ± 0.08 b	89.55 ± 0.40 b
	**OA 0.5**		9.14 ± 0.22 a	13.73 ± 0.12 b	7.58 ± 0.02 b	6.71 ± 0.06 b	89.99 ± 0.51 a
	**OA 1**		9.37 ± 0.25 a	13.63 ± 0.14 b	7.90 ± 0.07 a	6.98 ± 0.03 a	91.20 ± 0.34 a
	**Control**	6.82 ± 0.03 a	6.27 ± 0.23 b	16.61 ± 0.21 a	6.60 ± 0.04 b	6.09 ± 0.02 b	87.32 ± 0.45 a
35	**OA 0.1**	6.33 ± 0.05 b	6.32 ± 0.30 b	15.94 ± 0.37 b	6.25 ± 0.03 c	5.82 ± 0.03 c	85.27 ± 0.51 b
	**OA 0.5**	5.92 ± 0.08 c	6.81 ± 0.33 a	14.27 ± 0.26 c	6.68 ± 0.05 b	6.18 ± 0.04 b	85.66 ± 0.49 b
	**OA 1**	4.21 ± 0.03 d	6.72 ± 0.24 a	12.47 ± 0.13 d	7.00 ± 0.02 a	6.37 ± 0.03 a	86.87 ± 0.31 a

**Table 2:** The activity of the antioxidant enzymes (U min^−1^ g^−1^), ascorbate peroxidase (APX), catalase (CAT), and peroxidase (POD) in the flavedo and total phenolic content (mg 100 g^−1^) in the flavedo and juice from the control and oxalic acid (OA) treated trees at harvest (day 0) and after 35 days of storage at 10 °C in the 2019 experiment. Data are the mean ± SE of the four replicates. Different lowercase letters in the same column indicate significant differences (*p* <0.05) among treatments for each sampling date.

Storage	Treatment	APX	CAT	POD	TPC	
Days			Flavedo		Flavedo	Juice
	**Control**	1132.5 ± 42.5 d	772.0 ± 68.4 c	8595.0 ± 135.2 b	211.4 ± 5.1 d	64.0 ± 2.6 b
**0**	**OA 0.1**	1698.0 ± 36.7 c	1782.0 ± 107.4 b	8515.0 ± 305.7 b	276.8 ± 4.8 c	71.7 ± 5.7 b
	**OA 0.5**	1794. 0 ± 50.1 b	1743.0 ± 108.84 b	13,710.0 ± 251.6 a	321.6 ± 4.8 b	83.4 ± 6.7 a
	**OA 1**	2208.0 ± 43.5 a	2037.6 ± 164.3 a	14,330.0 ± 626.3 a	361.4 ± 5.1 a	92.3 ± 9.1 a
	**Control**	1997.6 ± 51.2 d	1064.8 ± 16.5 c	9888.9 ± 221.8 c	237.9 ± 3.2 d	71.2 ± 2.3 c
**35**	**OA 0.1**	2843.7 ± 59.2 c	1924.9 ± 101.9 b	9987.4 ± 165.3 c	294.5 ± 5.7 c	73.4 ± 2.4 c
	**OA 0.5**	3007.8 ± 78.3 b	1989.5± 77.3 b	14,125.4 ± 189.4 b	342.3 ± 6.1 b	89.5 ± 2.2 b
	**OA 1**	3505.2 ± 72.2 a	2556.2 ± 33.1 a	15,162.0 ± 199.5 a	375.1 ± 4.1 a	99.5 ± 1.7 a
**Storage**	**Treatment**	**APX**	**CAT**	**POD**	**TPC**	
**Days**			**Flavedo**		**Flavedo**	**Juice**
	**Control**	1132.5 ± 42.5 d	772.0 ± 68.4 c	8595.0 ± 135.2 b	211.4 ± 5.1 d	64.0 ± 2.6 b
**0**	**OA 0.1**	1698.0 ± 36.7 c	1782.0 ± 107.4 b	8515.0 ± 305.7 b	276.8 ± 4.8 c	71.7 ± 5.7 b
	**OA 0.5**	1794. 0 ± 50.1 b	1743.0 ± 108.84 b	13,710.0 ± 251.6 a	321.6 ± 4.8 b	83.4 ± 6.7 a
	**OA 1**	2208.0 ± 43.5 a	2037.6 ± 164.3 a	14,330.0 ± 626.3 a	361.4 ± 5.1 a	92.3 ± 9.1 a
	**Control**	1997.6 ± 51.2 d	1064.8 ± 16.5 c	9888.9 ± 221.8 c	237.9 ± 3.2 d	71.2 ± 2.3 c
**35**	**OA 0.1**	2843.7 ± 59.2 c	1924.9 ± 101.9 b	9987.4 ± 165.3 c	294.5 ± 5.7 c	73.4 ± 2.4 c
	**OA 0.5**	3007.8 ± 78.3 b	1989.5± 77.3 b	14,125.4 ± 189.4 b	342.3 ± 6.1 b	89.5 ± 2.2 b
	**OA 1**	3505.2 ± 72.2 a	2556.2 ± 33.1 a	15,162.0 ± 199.5 a	375.1 ± 4.1 a	99.5 ± 1.7 a

## Data Availability

Data is contained within the article.

## References

[1] Faostat (2019). Production Statistics. http://faostat.fao.org/site/567/DesktopDefault.aspx?PageID=567#ancor.

[2] Baldwin E.A., Bai J., Plotto A., Ritenour M.A. (2014). Citrus fruit quality assessment; producer and consumer perspectives. Stewart Postharvest Rev..

[3] Xavier S., Barbosa C., Barros D., Silva R., Oliveira A., Freitas R. (2007). Vitamin C antioxidant effects in hippocampus of adult Wistar rats after seizures and status epilepticus induced by pilocarpine. Neurosci. Lett..

[4] Parhiz H., Roohbakhsh A., Soltani F., Rezaee R., Iranshahi M. (2015). Antioxidant and Anti-Inflammatory Properties of the Citrus Flavonoids Hesperidin and Hesperetin: An Updated Review of their Molecular Mechanisms and Experimental Models. Phytother. Res..

[5] Gil-Izquierdo A., Riquelme M.T., Porras A.I., Ferreres F. (2004). Effect of the Rootstock and Interstock Grafted in Lemon Tree (*Citrus limon* (L.) Burm.) on the Flavonoid Content of Lemon Juice. J. Agric. Food Chem..

[6] Robles J., Botía P., Pérez-Pérez J. (2017). Sour orange rootstock increases water productivity in deficit irrigated ‘Verna’ lemon trees compared with Citrus macrophylla. Agric. Water Manag..

[7] Di Matteo A., Simeone G.D.R., Cirillo A., Rao M.A., Di Vaio C. (2021). Morphological characteristics, ascorbic acid and antioxidant activity during fruit ripening of four lemon (*Citrus limon* (L.) Burm. F.) cultivars. Sci. Hortic..

[8] Valero D., Serrano M. (2010). Postharvest Biology and Technology for Preserving Fruit Quality.

[9] Shimada M., Akamtsu Y., Tokimatsu T., Mii K., Hattori T. (1997). Possible biochemical roles of oxalic acid as a low molecular weight compound involved in brown-rot and white-rot wood decays. J. Biotechnol..

[10] Martínez-Esplá A., Zapata P.J., Valero D., García-Viguera C., Castillo S., Serrano M. (2014). Preharvest Application of Oxalic Acid Increased Fruit Size, Bioactive Compounds, and Antioxidant Capacity in Sweet Cherry Cultivars (Prunus aviumL.). J. Agric. Food Chem..

[11] Razavi F., Hajilou J. (2016). Enhancement of postharvest nutritional quality and antioxidant capacity of peach fruits by preharvest oxalic acid treatment. Sci. Hortic..

[12] García-Pastor M.E., Giménez M.J., Valverde J.M., Guillén F., Castillo S., Martínez-Romero D., Serrano M., Valero D., Zapata P.J. (2020). Preharvest Application of Oxalic Acid Improved Pomegranate Fruit Yield, Quality, and Bioactive Compounds at Harvest in a Concentration-Dependent Manner. Agronomy.

[13] Ding Z.-S., Tian S.-P., Zheng X.-L., Zhou Z.-W., Xu Y. (2007). Responses of reactive oxygen metabolism and quality in mango fruit to exogenous oxalic acid or salicylic acid under chilling temperature stress. Physiol. Plant.

[14] Liang C., Lv J., Jin M., Li H., Rao J. (2017). Effects of oxalic acid treatment on chilling injury, antioxidant capacity and energy status in harvested kiwifruits under low temperature stress. Acta Hort. Sin..

[15] Jin P., Zhu H., Wang L., Shan T., Zheng Y. (2014). Oxalic acid alleviates chilling injury in peach fruit by regulating energy metabolism and fatty acid contents. Food Chem..

[16] Wang Z., Cao J., Jiang W. (2016). Changes in sugar metabolism caused by exogenous oxalic acid related to chilling tolerance of apricot fruit. Postharvest Biol. Technol..

[17] Wang Q., Lai T., Qin G., Tian S. (2008). Response of Jujube Fruits to Exogenous Oxalic Acid Treatment Based on Proteomic Analysis. Plant Cell Physiol..

[18] Deng J., Bi Y., Zhang Z., Xie D., Ge Y., Li W., Wang J., Wang Y. (2015). Postharvest oxalic acid treatment induces resistance against pink rot by priming in muskmelon (*Cucumis melo* L.) fruit. Postharvest Biol. Technol..

[19] Martínez-Esplá A., Zapata P.J., Valero D., Martínez-Romero D., Díaz-Mula H.M., Serrano M. (2018). Preharvest treatments with salicylates enhance nutrient and antioxidant compounds in plum at harvest and after storage. J. Sci. Food Agric..

[20] Serna-Escolano V., Martínez-Romero D., Giménez M.J., Serrano M., García-Martínez S., Valero D., Valverde J.M., Zapata P.J. (2021). Enhancing antioxidant systems by preharvest treatments with methyl jasmonate and salicylic acid leads to maintain lemon quality during cold storage. Food Chem..

[21] Serna-Escolano V., Valverde J.M., García-Pastor M.E., Valero D., Castillo S., Guillén F., Martínez-Romero D., Zapata P.J., Serrano M. (2019). Pre-harvest methyl jasmonate treatments increase antioxidant systems in lemon fruit without affecting yield or other fruit quality parameters. J. Sci. Food Agric..

[22] Martínez-Esplá A., Serrano M., Martínez-Romero D., Valero D., Zapata P.J. (2019). Oxalic acid preharvest treatment increases antioxidant systems and improves plum quality at harvest and during postharvest storage. J. Sci. Food Agric..

[23] Razzaq K., Khan A.S., Malik A.U., Shahid M., Ullah S. (2015). Effect of oxalic acid application on Samar Bahisht Chaunsa mango during ripening and postharvest. LWT.

[24] Martínez-Esplá A., García-Pastor M.E., Zapata P.J., Guillen F., Serrano M., Valero D., Gironés-Vilaplana A. (2017). Preharvest application of oxalic acid improves quality and phytochemical content of artichoke (*Cynara scolymus* L.) at harvest and during storage. Food Chem..

[25] Barberis A., Cefola M., Pace B., Azara E., Spissu Y., Serra P.A., Logrieco A.F., D’Hallewin G., Fadda A. (2019). Postharvest application of oxalic acid to preserve overall appearance and nutritional quality of fresh-cut green and purple asparagus during cold storage: A combined electrochemical and mass-spectrometry analysis approach. Postharvest Biol. Technol..

[26] Serna-Escolano V., Serrano M., Valero D., Rodríguez-López M.I., Gabaldón J.A., Castillo S., Valverde J.M., Zapata P.J., Guillén F., Martínez-Romero D. (2020). Thymol Encapsulated into HP-β-Cyclodextrin as an Alternative to Synthetic Fungicides to Induce Lemon Resistance Against Sour Rot Decay. Molecules.

[27] Kvaratskhelia M., Winkel C., Thorneley R., Wenzl P., Patiño G.M., Chaves A.L., Mayer J.E., Rao I.M. (1997). Purification and Characterization of a Novel Class III Peroxidase Isoenzyme from Tea Leaves. Plant Physiol..

[28] Fawole O.A., Opara U.L. (2013). Effects of maturity status on biochemical content, polyphenol composition and antioxidant capacity of pomegranate fruit arils (cv. ‘Bhagwa’). S. Afr. J. Bot..

[29] Zheng X., Jing G., Liu Y., Jiang T., Jiang Y., Li J. (2012). Expression of expansin gene, MiExpA1, and activity of galactosidase and polygalacturonase in mango fruit as affected by oxalic acid during storage at room temperature. Food Chem..

[30] Sayyari M., Valero D., Babalar M., Kalantari S., Zapata P.J., Serrano M. (2010). Prestorage Oxalic Acid Treatment Maintained Visual Quality, Bioactive Compounds, and Antioxidant Potential of Pomegranate after Long-Term Storage at 2 °C. J. Agric. Food Chem..

[31] Li T., Shi D., Wu Q., Yin C., Li F., Shan Y., Duan X., Jiang Y. (2019). Mechanism of Cell Wall Polysaccharides Modification in Harvested ‘Shatangju’ Mandarin (*Citrus reticulate* Blanco) Fruit Caused by *Penicillium italicum*. Biomolecules.

[32] Ballester A.-R., Lafuente M.T., González-Candelas L. (2013). Citrus phenylpropanoids and defence against pathogens. Part II: Gene expression and metabolite accumulation in the response of fruits to Penicillium digitatum infection. Food Chem..

[33] Rojas C.M., Esenthil-Kumar M., Etzin V., Mysore K.S. (2014). Regulation of primary plant metabolism during plant-pathogen interactions and its contribution to plant defense. Front. Plant Sci..

[34] Yun Z., Gao H., Liu P., Liu S., Luo T., Jin S., Xu Q., Xu J., Cheng Y., Deng X. (2013). Comparative proteomic and metabolomic profiling of citrus fruit with enhancement of disease resistance by postharvest heat treatment. BMC Plant Biol..

